# Lightweight User Equipment-Side Detection of False Base Station Attacks Using a First-Order Markov Chain

**DOI:** 10.3390/s26134116

**Published:** 2026-06-29

**Authors:** Hoonyong Park, Vincent Abella, Ilsun You

**Affiliations:** 1 AUTOCRYPT Co., Ltd., Seoul 07241, Republic of Korea; hypark@autocrypt.io; 2 Department of Information Security, Cryptology, and Mathematics, Kookmin University, Seoul 02707, Republic of Korea; vincent@kookmin.ac.kr; 3 KMU Global Research Center for ICT Convergence Security, Kookmin University, Seoul 02707, Republic of Korea

**Keywords:** cellular network security, false base station, user equipment, Markov chain, anomaly detection, LTE, 5G

## Abstract

False base station (FBS) attacks exploit the attach window before the network authenticates to the device. Existing User Equipment (UE)-side detectors typically need either labeled attack data, which is scarce and does not generalize to unseen attacks, or models too heavy for the resource budget of a smartphone or embedded endpoint. This study presents a lightweight UE-side detector built on a first-order Markov chain over a four-tuple state of packet type, direction, message identifier, and access-network type. A single counting pass fits the 119 KB chain, and thresholds are derived from normal traffic, so no attack labels are consulted. The capture path requires root and Qualcomm modem diagnostic access. Attacks surface as low-probability transitions, rare field values, and anomalous pacing, fused into a per session verdict with per-message attribution. On 192 commercial, testbed, and public LTE and 5G captures, the detector flags 51 of 53 attacks at an F1 of 88.70% in leakage-free leave-one-session-out evaluation and 96.23% once calibration covers the scored sessions. In five-fold cross-validation its F1 of 86.21% trails the strongest supervised baselines by margins that are not statistically significant, and it records the lowest latency (0.46 ms) and smallest working set (8.8 MB) among the eleven detectors benchmarked.

## 1. Introduction

Cellular access protocols leave one side of the attach procedure unauthenticated. The subscriber device proves its identity to the operator core, but the operator radio is not authenticated back to the device until the security context is established. A false base station (FBS) abuses that gap. The FBS transmits a reference signal whose received power exceeds that of the genuine cell on the same carrier and draws nearby devices into its coverage. Smartphones and Internet-of-Things (IoT) endpoints are exposed most sharply. The share of memory, compute, and energy a host can give to a continuously running defensive monitor is bounded by the rest of the device’s workload. A detector that exceeds that share displaces other on-device subsystems or cannot run continuously at all.

These deployments are not hypothetical. Li et al. [1] uncovered false base stations operating at scale in the wild. The root cause persists because the bootstrapping messages and base station broadcasts are not authenticated, and the device cannot distinguish a legitimate cell from a fake one [2]. From that position an attacker harvests permanent identifiers and device capabilities, downgrades security, and injects reject loops in the window before the security context is active [3,4,5]. The exposure carries into 5G, where targeted identity tracking survives the move to encrypted subscription identifiers, and false base stations remain a primary concern for current detection research [6,7,8]. Detection approaches that operate on the User Equipment (UE) itself fall into three families, distinguished by the data source they consume. Supervised deep-learning detectors over dissected signaling messages, such as the LSTM-and-graph FBSDetector of Mubasshir et al. [9], need substantial labeled attack collections that are scarce and do not generalize to unseen attacks. Modem-diagnostic monitors such as PHOENIX [10] bind their capture path to the vendor diagnostic interface and require root. Specification-based and platform-level detectors such as CellGuard [11] avoid attack labels but rest on manually authored rules or a constrained deployment surface. Section 3 surveys these families in full. Each path leaves a coverage gap on a device that has no labeled attack history and only a small compute and memory share to spare for a continuous monitor.

This study addresses that gap with a detector that fits within the memory and compute budget of typical UE and IoT hardware. Protocol behavior is represented as a first-order Markov chain over a categorical state derived from layer-3 message metadata, and an anomaly score combines information-theoretic transition surprise with conditional field-value rarity and per-state burst rate. The chain parameters and every decision threshold are estimated from normal traces alone, so the pipeline consults no attack labels at any stage. Each verdict completes in sub-millisecond time and carries a per-row attribution that can be inspected on the device without sending captures off the handset. The capture pipeline requires root and Qualcomm modem diagnostic access on the host device, which confines the prototype to rooted Qualcomm-modem smartphones and IoT-class endpoints with vendor diagnostic permission.

The contributions of this study are summarized as follows.

- A first-order Markov chain detector that represents each layer-3 message as a four-tuple state of packet type, signaling direction, message identifier, and access-network type and scores every session through three fused channels of information-theoretic transition surprise, conditional field-value rarity, and per-state burst rate, so that reordered procedures, rare field values, and attack-induced pacing each raise an alert.
- A normal-only calibration procedure that fits the chain in a single counting pass over normal traces and derives every decision threshold from normal-score statistics through an extreme-value tail rule, consulting no attack labels at any stage. The single-pass estimator yields a compact model and per-row attribution that names the offending transition and field value, supporting on-device forensics within a continuous-monitoring budget.
- An empirical comparison against lightweight supervised and unsupervised baselines on a real-world LTE and 5G FBS dataset, paired with a hardware-constrained deployment benchmark that measures each detector under a smartphone and IoT-gateway-class memory and compute budget.

Section 2 reviews the relevant slice of the cellular protocol stack, enumerates FBS attack categories, and introduces the Markov-chain anomaly-detection foundation. Section 3 surveys prior UE-side detection efforts. Section 4 introduces the proposed detector, including the state representation; the row, window, and session scoring rules; and the threshold calibration procedure. Section 5 documents the dataset, preprocessing, evaluation protocol, and reports detection effectiveness alongside the resource profile and attribution analyses. Section 6 situates the approach within the design space of lightweight UE-side detectors, and Section 7 concludes.

## 2. Background

### 2.1. False Base Station Attacks

A false base station (FBS) is an attacker-operated cell that transmits at higher received power than the legitimate cell on the same carrier, wins the device’s cell selection, and then operates in the window before the security context is active, where signaling carries neither integrity nor confidentiality. Research on FBS attacks has progressed from ad hoc International Mobile Subscriber Identity (IMSI) catchers to systematic analyses of the LTE and 5G control plane. Shaik et al. [3] demonstrated practical privacy and availability attacks on LTE from a low-cost FBS, and Li et al. [1] later found such cells operating at scale in the wild. Model-checking frameworks then systematized the surface, with LTEInspector [5] and 5GReasoner [12] uncovering authentication, paging, and downgrade flaws across 4G and 5G. More recent work extends the surface into 5G through capability harvesting [4], identity tracking that survives the encrypted Subscription Concealed Identifier (SUCI) [6], bidding-down of security algorithms [13], and signal overshadowing [14]. A recent survey collects this progression for 5G and 6G [7]. Six recurring attack patterns run through this literature.

Identity exposure answers identity requests before authentication to harvest the subscriber or equipment identity and track the device [3]. Rejection-based denial of service injects pre-security reject messages that force repeated re-attachment and keep the device on the FBS [5]. Security downgrade and manipulation steers the security-mode negotiation toward null algorithms or strips NAS security headers to weaken later traffic [4,13]. Authentication attacks replay or relay authentication exchanges and drive UE counters into terminal states [12]. Cell and Radio Access Network (RAN) spoofing and control-plane hijacking forges system-information frames and delivers messages out of order to hijack paging, handover, or reconfiguration [14]. Resource exhaustion sustains pre-authentication signaling that keeps the modem active and drains the battery [3].

**Figure 1:**
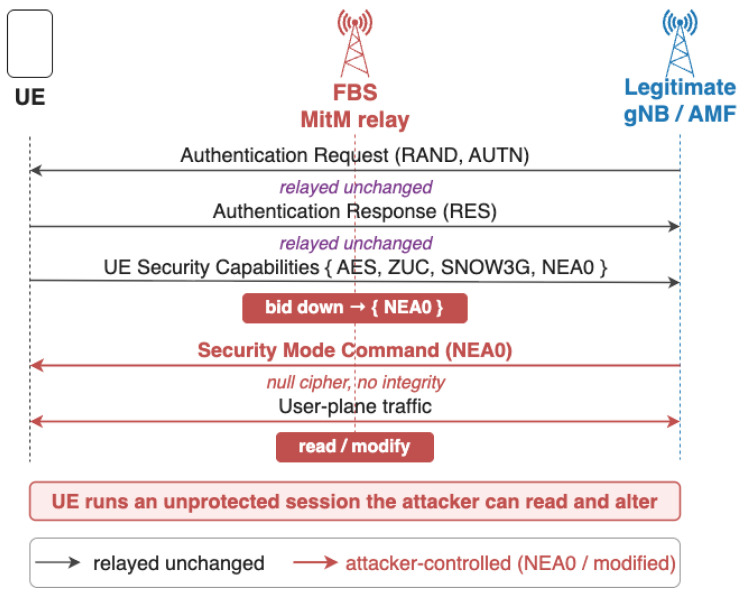
A man-in-the-middle false base station relay that forwards authentication unchanged, bids the security context down to a null cipher, and modifies the resulting user-plane traffic.

Figure 1 traces a recent, high-impact case that chains several of these patterns. A man-in-the-middle relay forwards the authentication exchange unchanged, bids the negotiated security algorithms down to the null cipher, and then modifies the resulting user-plane traffic [5,13].

Other FBS-relevant identifiers used in later sections are the International Mobile Equipment Identity (IMEI), the Tracking Area Update (TAU) procedure, the System Information Block (SIB) broadcast, the Public Land Mobile Network (PLMN) code, and the Tracking Area Code (TAC).

These six patterns define the threat scope used in the experimental sections, which is restricted to misbehavior observable in the unprotected pre-security window. The in-scope Security downgrade and Cell and RAN spoofing categories therefore cover only the plaintext, pre-security manifestations of their underlying mechanisms. Three 5G-specific variants confine their evidence to protected or ciphered content and sit outside this scope, noted as bounded-generality limitations. Null-scheme SUCI exposure [15] turns on a field the dissector cannot expand. Authentication-and-key-agreement bidding-down [13] manifests in authentication parameters outside the scored constraint fields rather than in plaintext message order. Ciphered-frame overshadowing [12,14] carries its payload inside encrypted frames.

### 2.2. NAS and RRC Layer-3 Signaling

The attacks of Section 2.1 all unfold in the layer-3 control plane, so that is the layer the detector examines. Layer-3 signaling splits into two families: Radio Resource Control (RRC) on the air interface, which handles bearer setup, security-mode activation, and mobility [16,17], and Non-Access-Stratum (NAS) signaling, which rides over RRC to the core for attach or registration, authentication, and session management [18,19]. A normal session proceeds through idle-mode cell selection, connection setup, authentication, and activation of the security context, after which traffic is integrity- and confidentiality-protected. Frames before the Security Mode Command are not, and this pre-secured window is the surface FBS adversary’s target. The 5G registration procedure follows the same partition with renamed messages, so the same window applies to both access generations.

Layer-3 signaling is also the only view of this window the device itself can read. The dissected NAS and RRC messages are exposed to the UE through its modem diagnostic interface, whereas physical-layer and core-side measurements are not, which makes layer-3 signaling the natural observation surface for the on-device detector of Section 4.

### 2.3. Markov-Chain Anomaly Detection

The layer-3 stream of Section 2.2 is a sequence of discrete, categorical protocol messages, and the FBS attacks of Section 2.1 perturb that sequence by reordering messages or carrying rare field values, so a model of the normal transition structure can flag both. Sequence-based anomaly detection has a long history in intrusion detection, from short-sequence system-call modeling [20] to recent lightweight Markov-chain detection at the network edge [21]. A first-order Markov chain over an observable categorical state provides a transparent generative model with a closed-form maximum likelihood estimator, which reduces fitting to a single counting pass and supports per-step attribution of anomaly scores. These properties match an on-device monitor that must surface localized protocol anomalies without uploading captures off the handset.

## 3. Related Work

UE-side detection approaches differ in data source, detection method, label requirements, and host-privilege requirements. Table 1 summarizes prior systems and the proposed detector along these dimensions. The supervised LSTM-and-graph FBS detector of Mubasshir et al. [9] and the quanvolutional neural-network detector of Pawana et al. [22] both depend on labeled attack data at training time, which is scarce and does not generalize to previously unseen behavior. FBSDetector itself runs in a small on-device footprint [9], so for the supervised family the labeled-data requirement, rather than model size, is the binding constraint. CellGuard [11] runs on non-jailbroken iOS devices and flags potential false base stations by checking the observed cell against Apple’s cell location database. A second body of effective detectors operates off the device and is complementary to the UE-side setting considered here: network-side machine learning on Reference Signal Received Power (RSRP) features [23], the specification-based SMDFbs detection function deployed in the radio access network [24], and the fleet-based SeaGlass [25], which aggregates readings from vehicle-mounted scanners for city-wide IMSI-catcher detection.

Software-defined-radio methodologies require external radio and are not deployable on a stock handset, so they fall outside the on-device setting. The proposed detector instead targets a smartphone- or IoT-gateway-class host through the on-device monitor of [26].

Another branch of UE-side work taps the modem diagnostic stream. PHOENIX [10] compiles Past-time Linear Temporal Logic invariants against MobileInsight-sourced RRC and NAS frames, and SnoopSnitch [27] watches Qualcomm DIAG output for radio-frequency channel changes and missing ciphering indicators. Both depend on root and vendor-supplied diagnostic permits. Earlier rule-based monitors such as IMSI-Catch Me [28] and AIMSICD [29] encode small heuristic catalogs over baseband indicators and inherit the same access constraint. CellDAM [30] runs a user-space state-dependent checker over the 5G data plane. The Markov chain instead learns a transition-and-field-rarity distribution from normal traces, flagging any rare protocol behavior the calibration data did not contain without enumerating the attack catalog ahead of time.

**Table 1:** UE-side false base station detection approaches and the proposed system.

System	Data Source	Method	Labels	5G	Privilege
Markov chain (proposed)	NAS/RRC traces	First-order Markov chain	No	Yes	Root, DIAG
FBSDetector [9]	NAS/RRC traces	LSTM and graph	Yes	No	Root
QCNN-FBS [22]	NAS/RRC traces	Quanvolutional NN	Yes	Yes	Root
CellGuard [11]	Apple cell DB	Database anomaly detection	No	Yes	None
CellDAM [30]	5G data plane	State-dependent checking	No	Yes	User space
PHOENIX [10]	NAS/RRC traces	PLTL runtime verification	No	No	Root, DIAG
IMSI-Catch Me [28]	Cell params, paging	Rule-based heuristics	No	No	Root
AIMSICD [29]	LAC, CID, cipher flags	Rule-based heuristics	No	No	Root
SnoopSnitch [27]	Qualcomm DIAG	Rule-based heuristics	No	No	Root, DIAG

As the method and label columns of Table 1 show, existing UE-side detectors reach their model by one of two routes. Supervised detectors such as FBSDetector learn from data but require labeled attacks, which are scarce and do not generalize to unseen behavior. Rule- and specification-based monitors such as PHOENIX, CellDAM, and SnoopSnitch need no labels but rely on hand-authored invariants, rules, or heuristics that must enumerate misbehavior in advance. No prior UE-side detector learns its model from unlabeled normal traffic alone, requiring neither attack labels nor hand-written rules. This study fills that label-free, learning-based corner with per-message attribution and a footprint small enough to run continuously on the device. The proposed chain, QCNN-FBS, CellGuard, and CellDAM also span 5G, whereas the supervised FBSDetector and the runtime-verification PHOENIX remain limited to 4G.

## 4. Proposed System

**Figure 2:**
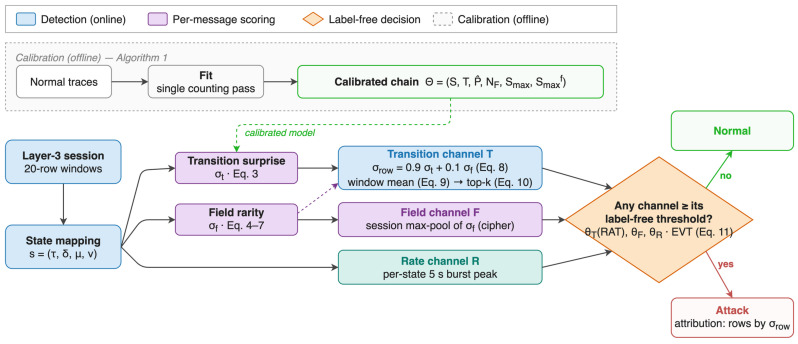
Detection pipeline that maps a layer-3 capture session to per-channel scores and a verdict, with the offline calibration of Algorithm 1 supplying the calibrated chain to the scoring stages.

The proposed detector is a single pipeline, shown end to end in Figure 2. An offline calibration pass fits a first-order Markov chain on normal layer-3 traces alone and consults no attack labels. At detection time the calibrated chain scores the capture session in one forward pass. Each message is mapped to a categorical state and scored for transition surprise and field-value rarity against the calibrated chain, the per-message scores are pooled over 20-row windows into a session-level score per channel, and the session is flagged as an attack when any channel exceeds its label-free threshold. The messages of a flagged session are ranked to attribute the verdict. In Figure 2, the offline calibration lane is separated from the online detection lane. The three scoring channels are color-coded, with the transition channel in blue, the field channel in purple, and the rate channel in teal. The two verdict outcomes appear as normal in green and attack in red.

### 4.1. Adversarial Model

The proposed detector runs on the device and sees only the dissected layer-3 message stream of one cellular session at a time, produced by the on-device monitor of [26] from the Qualcomm modem diagnostic interface. It has no access to physical-layer or MAC-layer measurements, operator-side core signaling, or the cryptographic verdicts the modem computes internally. Detection therefore shares its observation surface with the attacker, the unprotected exchange that precedes activation of the security context.

**Figure 3:**
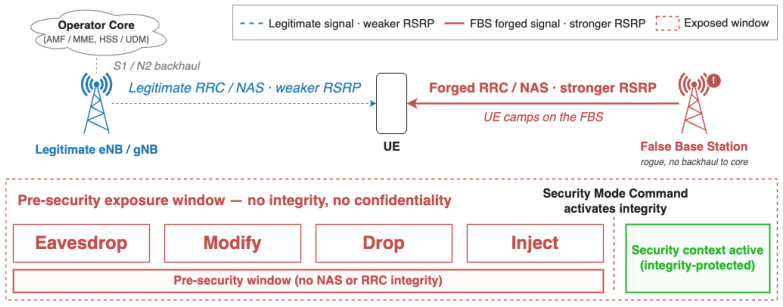
Adversarial model in which a false base station overshadows the legitimate cell at higher received power and acts as an active Dolev–Yao attacker on RRC and NAS within the pre-security exposure window that the Security Mode Command closes by activating integrity protection.

Figure 3 shows the setting. Following established FBS threat modeling [3,5], the adversary is an FBS that broadcasts at higher received power than the legitimate cell on the same carrier, wins the UE’s cell selection, and acts as an active Dolev–Yao attacker on RRC and NAS until the Security Mode Command activates the integrity context. Within that window it can eavesdrop, modify, drop, or inject any frame. The 3GPP cryptographic primitives are taken to be unbroken: SIM subscription keys cannot be extracted, authentication-and-key-agreement responses cannot be forged without them, and an integrity-protected NAS session is no longer subject to tampering. The exposed interval therefore runs from idle-mode cell selection to security-context activation, and reopens whenever a mobility procedure rebuilds that context from scratch. Inter-MME or inter-AMF handover, idle-mode tracking-area update after timer expiry, and re-attach after radio link failure derive fresh keys and re-execute the Security Mode Command, so they reopen the pre-secured window. Intra-MME handover with key forwarding and RRC connection resume preserve the existing context and do not. This security-context handling is specified for LTE in 3GPP TS 33.401 [31], clause 7, and for 5G in 3GPP TS 33.501 [32], clauses 6.7 and 6.9.

### 4.2. Protocol State Representation

Each layer-3 message in a dissected trace is mapped to a state s=(τ,δ,μ,ν). The four components identify the message. The component τ identifies the packet type, with values drawn from RRC, NAS, or composite NAS + RRC frames. The component δ identifies the signaling direction, with values uplink, downlink, or unspecified. The component μ identifies the human-readable message name extracted from the dissected trace, such as RRC Connection Setup, Security Mode Command, or Attach Request. The component ν identifies the access-network type, with values LTE, 5G, or COMMON. The COMMON value applies to messages that carry no network-specific markers, which include certain broadcast and paging frames shared by both LTE and 5G.

The state deliberately excludes message pacing. Embedding an inter-arrival time bucket as a fifth state component would multiply the state space by the bucket count and tie the model to deployment-dependent timing cut points. The state therefore stays purely protocol-identifying, and pacing is monitored through a separate burst-rate channel introduced in Section 4.6, which counts messages per state inside a sliding five-second window against caps calibrated from normal traffic.

The access-network slot ν serves two purposes. It distinguishes equivalently named messages whose underlying procedure differs across LTE and 5G, which prevents collisions in the learned distribution. It also enables a single calibrated model to operate on traces that span pure LTE deployments, 5G Standalone (SA) deployments served by an open5gs core and an srsRAN gNB, and 5G Non-Standalone (NSA) deployments in which LTE and 5G messages interleave. Cross-network transitions in NSA traces are recorded as ordinary transitions in the same Markov chain, which removes the need for separate models per access generation.

### 4.3. Probabilistic State Machine

The Markov chain is built by a single, non-iterative calibration pass over the normal traces in the training split. The pass produces two outputs: the set of observed states S and the maximum-likelihood estimate of the conditional transition probability $\hat{P}\left(s_{j}|s_{i}\right)$ for each ordered pair $\left(s_{i},s_{j}\right)$ between them. No loss is minimized and no gradient step is taken, which is why a 0.23 s wall-clock fit is sufficient to populate the chain (Section 5.6).

During the pass, the detector maintains a count $n(s_{i},s_{j})$ for each ordered pair of states observed in consecutive positions of the calibration traces, together with a per-state outgoing total $n\left(s_{i}\right)=\sum_{s_{j}\in S}n\left(s_{i},s_{j}\right)$. The maximum likelihood estimate of the conditional transition probability is

(1) $$\hat{P}\left(s_{j}|s_{i}\right)\;=\;\frac{n(s_{i},s_{j})+\alpha }{n(s_{i})+\alpha \;|S|},$$

where |S| is the cardinality of the state set S and fixes the size of the smoothing support. The smoothed estimate is a proper conditional distribution because the numerator summed over destinations equals the denominator, so $\sum_{s_{j}\in S}\hat{P}\left(s_{j}|s_{i}\right)=1$ for every source state $s_{i}$. The additive smoothing constant $\alpha =10^{-4}$ is chosen small relative to typical transition counts while still ensuring finite log-scores for transitions that occur in deployment but were absent during calibration. The sensitivity analysis of Section 5.4 varies α over four orders of magnitude and finds every verdict unchanged, so the constant is not a tuned parameter. The calibration routine also computes a maximum surprise constant

(2) $$S_{\max}\;=\;\max_{(s_{i},s_{j})\in T}\left[-\log_{2}\hat{P}\left(s_{j}|s_{i}\right)\right]$$

over the set of transitions T that received non-zero counts. The constant $S_{\max}$ normalizes the transition surprise into a bounded interval, which simplifies threshold calibration and stabilizes the aggregation rule introduced below. Because $S_{\max}$ is the largest surprise over the observed transitions in T, the first-case score $\min\left(\left(-\log_{2}\hat{P}\left(s_{j}|s_{i}\right)\right)/S_{\max},\;1\right)$ of Equation (3) lies in [0, 1] for every observed transition, and its cap at 1 never binds there.

**Figure 4:**
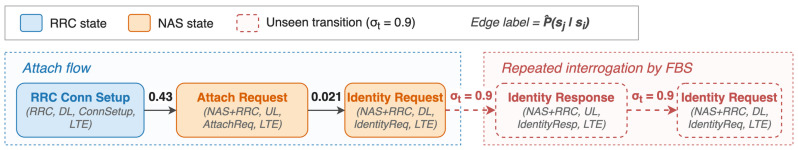
Measured excerpt of the calibrated Markov chain on a representative identity-catching attack capture.

Figure 4 shows a measured excerpt from a representative identity-catching attack capture, scored for the chain calibrated on the 139 normal captures. The edge from Attach Request to Identity Request is rare but legal since the network may request an identity during initial attach before a security context exists, and the calibrated chain assigns it $\hat{P}=0.021$. The false base station then repeats the interrogation to harvest both the IMSI and the IMEI. The edge from the Identity Response back to a further Identity Request appears in no normal capture, so each repetition scores the flat unseen-transition surprise of Equation (3), and the transition channel crosses its calibrated threshold. Pre-security Identity Requests occur in 12 of the 53 attack captures and in 8 of the 139 normal captures, where a single request at initial attach is legal. The repeated-interrogation edge occurs in 3 attack captures and in no normal capture. The same capture is used for the attribution example of Section 5.7.

### 4.4. Per-Row Anomaly Score

Two complementary signals contribute to the per-row anomaly score. The first is the transition-surprise term $\sigma_{t}\left(s_{i}\to s_{j}\right)$, which scores how unexpected the ordered transition from source state $s_{i}$ to destination state $s_{j}$ is under the calibrated chain conditions. It is computed by

(3) $$\sigma_{t}\left(s_{i}\to s_{j}\right)\;=\;\left\{\begin{array}{cc} \min\;\left(\left(-\log_{2}\hat{P}\left(s_{j}|s_{i}\right)\right)/\;S_{\max},\;1\right) & (s_{i},s_{j})\in T\\ 0.9 & s_{j}\in S,\;\left(s_{i},s_{j}\right)\notin T\\ 1.0 & s_{j}\notin S \end{array}\right.$$

The first case applies when the transition was observed during calibration. The second case applies when the destination state was observed but the specific transition was not. The third case applies when the destination state itself was unobserved during calibration. The fixed constants 0.9 and 1.0 distinguish the two unseen regimes and bound the score below the saturation point of the smoothed logarithm. Because $S_{\max}$ of Equation (2) normalizes by the rarest observed transition, a legal-but-rare observed transition can score above the flat 0.9 of an unseen transition, an intended ordering since a transition barely attested in calibration is no less surprising than a merely novel one. The sensitivity analysis of Section 5.4 varies the unseen-transition constant over 0.8, 0.9, and 1.0 and finds that the result moves by at most one F1 point. At the first row of a session, where the source state $s_{i}$ has no predecessor, the row contributes $\sigma_{t}=0$ if $s_{j}\in S$ and $\sigma_{t}=1$ otherwise, so a session that opens on a previously unseen state is still penalized.

The second is the field-rarity term $\sigma_{f}\left(v|f,s_{i}\to s_{j}\right)$, which scores how unusual the value *v* of field *f* is in its protocol context for the calibrated chain. The detector tracks a curated set F of nine protocol-relevant constraint fields drawn from the dissected layer-3 record (Table 2): the LTE-RRC ciphering and integrity algorithms, the NAS-EPS EMM cause and identity-type codes, the RRC release cause, the cell-barred flag, the MCC and MNC codes, and the minimum receive level $q_{\mathrm{rxlevmin}}$. For a populated field *f* carrying value *v* in a row mapped to the transition $s_{i}\to s_{j}$, the value’s probability is estimated with open-vocabulary additive smoothing in each of two contexts,

(4) $${\hat{p}}_{c}\left(v|f\right)\;=\;\frac{n_{c}\left(v|f\right)+\lambda }{n_{c}\left(f\right)+\lambda \;\left(K_{c}\left(f\right)+1\right)},$$

where the context *c* is either the transition $s_{i}\to s_{j}$ or the destination state $s_{j}$, $n_{c}\left(v|f\right)$ counts occurrences of the value in that context during calibration, $n_{c}\left(f\right)$ is the populated total, $K_{c}\left(f\right)$ is the number of distinct values seen, the +1 reserves probability mass for an unseen value, and λ=0.1. The two contexts are blended by transition support,

(5) $$\hat{p}\left(v|f,s_{i}\to s_{j}\right)\;=\;w\;{\hat{p}}_{\mathrm{edge}}\left(v|f\right)+\left(1-w\right)\;{\hat{p}}_{\mathrm{state}}\left(v|f\right),\;w=\frac{n_{\mathrm{edge}}\left(f\right)}{n_{\mathrm{edge}}\left(f\right)+C},$$

with the back-off constant C=2. A well-sampled transition keeps its own estimate and a sparse transition defers to the destination state, so a null cipher on a rarely traversed edge still registers against the well-sampled Security Mode Command state, while a value that is legal at the state level stays unflagged. The field surprise of the blended estimate of Equation (5) is then

(6) $$\sigma_{f}\left(v|f,s_{i}\to s_{j}\right)\;=\;\min\;\left(\left(-\log_{2}\hat{p}\left(v|f,s_{i}\to s_{j}\right)\right)/\;S_{\max}^{f},\;1\right),$$

where $S_{\max}^{f}$ is a field-specific normalizer set to the largest surprise the calibrated model can assign to any field value, namely, $-\log_{2}$ of the smallest reserved unseen-value mass across all calibrated contexts. The per-row field score takes the maximum over the set $F_{r}$ of fields populated in row *r*,

(7) $$\sigma_{f}^{\max}\;=\;\max_{f\in F_{r}}\sigma_{f}\left(v|f,s_{i}\to s_{j}\right),$$

rather than their average, so a single anomalous field, such as a null cipher in an otherwise normal row, is not diluted across benign fields.

The two terms are combined into a single per-row score by a weighted sum,

(8) $$\sigma_{\mathrm{row}}\;=\;w_{t}\;\sigma_{t}+w_{f}\;\sigma_{f}^{\max},\;w_{t}=0.9,\;w_{f}=0.1.$$

The dominant transition weight reflects the fact that most FBS attacks perturb protocol message order. The non-zero field weight retains a per-row contribution from rare field values. The sensitivity analysis of Section 5.4 finds the results identical for any transition weight between 0.7 and 0.9 and degraded only when the field term is removed entirely. Field-value anomalies are additionally monitored by a dedicated field channel with its own threshold (Section 4.6), so the per-row weight is a secondary path for field evidence rather than its only path.

### 4.5. Window and Session Scores

The detector consumes dissected layer-3 messages in 20-row windows, the cadence at which the on-device monitor reads them out of the modem capture path. The window size is therefore a property of the data acquisition pipeline rather than a tuning parameter of the model, and the sensitivity analysis of Section 5.4 confirms the results are unchanged for windows of 30 to 50 rows. Within each window of N=20 rows, the window score is the mean of the per-row scores,

(9) $$\sigma_{\mathrm{win}}\;=\;\frac{1}{N}\sum_{m=1}^{N}\sigma_{\mathrm{row}}^{\left(m\right)}.$$

The unit of decision is the capture session. A session of *M* rows yields ⌈M/N⌉ consecutive windows, and the session-level transition score is the mean of its k=3 highest window scores,

(10) $$T\;=\;\frac{1}{k}\sum_{j=1}^{k}\sigma_{\mathrm{win}}^{\left[j\right]},$$

where $\sigma_{\mathrm{win}}^{\left[j\right]}$ denotes the *j*-th largest window score of the session. An attack occupies a contiguous minority of a long capture, so a mean over all windows dilutes the anomalous segment while a single maximum is sensitive to one noisy window. The top-*k* mean requires the anomaly to be sustained across several windows before the session score rises. The sensitivity analysis of Section 5.4 shows both alternatives degrade, with k=1 costing 5.5 F1 points and k=5 costing 3.2.

### 4.6. Threshold Calibration and Decision Rule

The chain is fitted only on normal traffic, and every decision threshold is likewise computed from the training normals alone, so the pipeline consults no attack labels at any stage. The detector fuses three scoring channels, each carrying its own label-free threshold. The transition channel score *T* is the top-*k* window mean of Equation (10), tested against a threshold $\theta_{T}$. The field channel score *F* max-pools the state-conditional field surprise of Equations (4)–(6) for the ciphering-algorithm field over the whole session, tested against a threshold $\theta_{F}$, which isolates field-only attacks such as a null-cipher Security Mode Command from the sequence evidence. The burst-rate channel records the per-state message count *R* inside a sliding five-second window and tests each per-state peak against a calibrated cap $\theta_{R}$, which monitors the pacing dimension the state representation deliberately excludes (Section 4.2). The five-second width is grounded in signaling timing rather than chosen heuristically. It spans several discontinuous-reception paging cycles, which TS 36.304 [33] bounds at 0.32 to 2.56 s, so benign periodic paging is averaged rather than flagged. It also sits below the 10-s NAS retry timer T3411 of TS 24.301 [18], so a single benign retry contributes to at most one window while an attack flood fills consecutive windows. A session is declared an attack when any channel crosses its threshold, that is, when $T\geq \theta_{T}$, $F\geq \theta_{F}$, or some per-state peak in *R* exceeds $\theta_{R}$.

Each channel threshold is derived from the distribution of that channel’s scores over the training-normal sessions. The rule is a peaks-over-threshold extreme-value estimate. Exceedances above the 80th percentile *u* of the normal scores are fit with a generalized Pareto distribution, and the threshold is placed at the quantile whose exceedance probability under normal traffic conditions is one percent,

(11) $$\theta \;=\;u+\frac{\hat{\beta }}{\hat{\xi }}\left[{\left(\zeta_{u}/p\right)}^{\hat{\xi }}-1\right],\;p=0.01,$$

where $\hat{\xi }$ and $\hat{\beta }$ are the fitted shape and scale and $\zeta_{u}$ is the empirical exceedance fraction. When fewer than 12 exceedances are available for a stable fit, the rule falls back to the maximum of the 95th percentile and μ+κσ, where μ and σ are the mean and standard deviation of those scores and the multiplier κ is 2 for the transition channel, 3.5 for the field channel, and 4 for the per-state rate caps. The per-state rate caps additionally carry a fixed floor of 20 messages per five-second window, which guards against degenerate caps on states the calibration data visits rarely. The transition-channel threshold $\theta_{T}$ is calibrated separately per radio access technology (RAT), one value for the LTE captures and one for the 5G captures since the two radio generations produce different normal score levels, with a pooled threshold as the fallback when a population is too small. The sensitivity analysis of Section 5.4 quantifies the contribution of the tail estimate: replacing it with the fallback rule alone costs three F1 points, the largest effect among the threshold-calibration constants.

**Figure 5:**
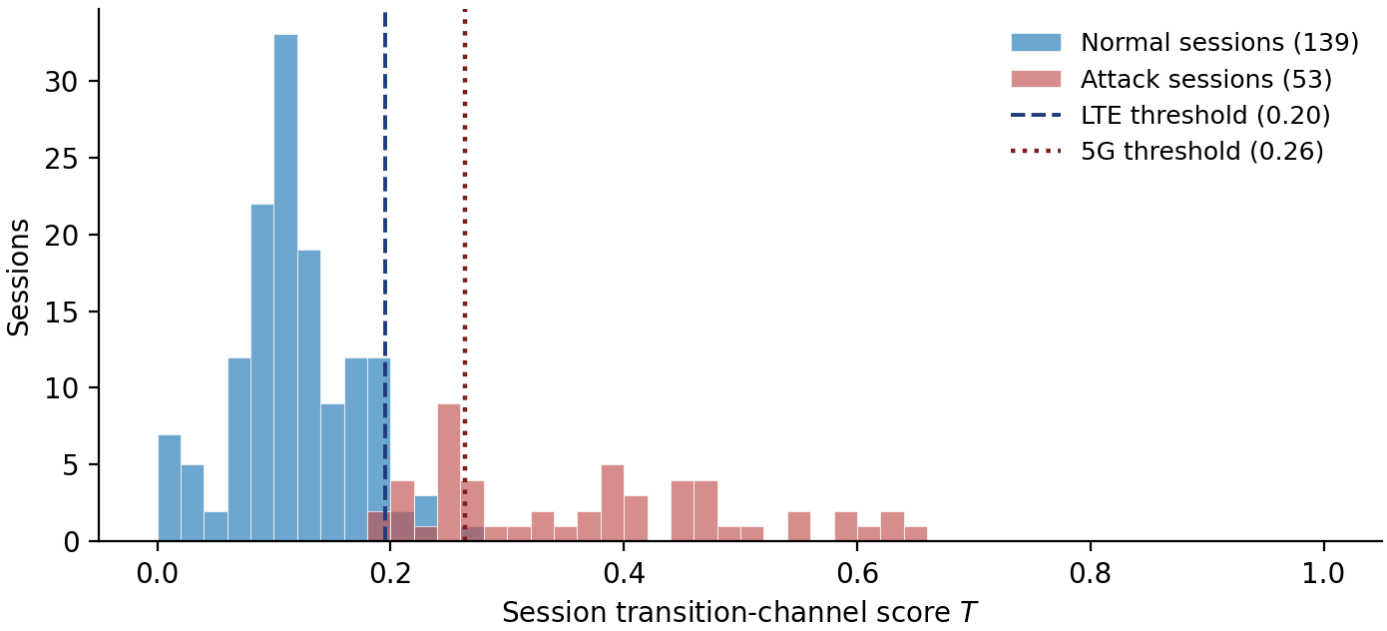
Per-session transition-channel scores in the deployment calibration, grouped by ground-truth class, with the label-free LTE and 5G thresholds shown as vertical dashed lines.

Figure 5 shows the empirical separation, with per session transition-channel scores grouped by ground-truth class and the calibrated label-free LTE and 5G thresholds drawn as vertical lines. Normal sessions concentrate at low scores, and attack sessions spread toward high scores. The two populations overlap in a narrow band around the thresholds, which is where the false positives and the missed attacks of Section 5 live, and the label-free rule places each threshold at the boundary of the normal mass without consulting a single attack label.

### 4.7. Algorithm Summary

Algorithm 1 formalizes the calibration of Section 4.2, Section 4.3 and Section 4.4. It estimates the state set S, transition set T, smoothed probabilities $\hat{P}$, field-value counts $N_{F}$, and the normalizers $S_{\max}$ and $S_{\max}^{f}$ from normal traces alone, consulting no attack labels. The accessor r.x reads field *x* from a dissected row, so s←(r.τ,r.δ,r.μ,r.ν) forms the four-tuple state. These six components are the entire model that scoring reads.
**Algorithm 1** Markov-chain calibration over normal layer-3 traces, producing the state set and transition probabilities used at scoring time**Require:** Normal training set $D_{N}$ of dissected traces; smoothing constants α,λ; constraint-field set F
**Ensure:** Calibrated chain $\Theta =(S,T,\hat{P},N_{F},S_{\max},S_{\max}^{f})$
  1:**procedure** Fit($D_{N},\alpha ,\lambda ,F$)  2:      S,T←∅; $n\left(\cdot ,\cdot \right),n\left(\cdot \right),N_{F}\leftarrow 0$  3:      **for** each trace $X\in D_{N}$ **do**  4:            $s_{\mathrm{prev}}\leftarrow \mathrm{undefined}$  5:            **for** each row r∈X in temporal order **do**  6:                   s←(r.τ,r.δ,r.μ,r.ν)  7:                   S←S∪{s}  8:                   **for** each f∈F with populated value *v* in *r* **do**  9:                         increment the per-state count of (v,f) at *s* in $N_{F}$10:                 **end for**11:                 **if** $s_{\mathrm{prev}}$ is defined **then**12:                       $n(s_{\mathrm{prev}},s)+=1$; $n(s_{\mathrm{prev}})+=1$13:                       $T\leftarrow T\cup \{\left(s_{\mathrm{prev}},s\right)\}$14:                       **for** each f∈F with populated value *v* in *r* **do**15:                             increment the per-transition count of (v,f) at $\left(s_{\mathrm{prev}},s\right)$ in $N_{F}$16:                       **end for**17:                 **end if**18:                 $s_{\mathrm{prev}}\leftarrow s$19:          **end for**20:    **end for**21:    Compute $\hat{P}\left(s_{j}|s_{i}\right)$ via Equation (1)               ▹ additive smoothing22:    $S_{\max}\leftarrow \max_{(s_{i},s_{j})\in T}\left[-\log_{2}\hat{P}\left(s_{j}|s_{i}\right)\right]$23:    $S_{\max}^{f}\leftarrow$ largest $-\log_{2}$ of the reserved unseen-value mass over all contexts in $N_{F}$24:    **return** $\Theta =(S,T,\hat{P},N_{F},S_{\max},S_{\max}^{f})$25:**end procedure**


Algorithm 2 states the detect path as a single forward pass with no further training, diagrammed in Figure 2. Each row is mapped to its state and scored for transition surprise $\sigma_{t}$ (Equation (3)) and field rarity $\sigma_{f}^{\max}$ (Equation (7)), combined into the per-row score $\sigma_{\mathrm{row}}$ (Equation (8)). Window means (Equation (9)) and top-*k* pooling (Equation (10)) give the session transition score *T*, the field channel *F* max-pools the cipher-field surprise, and the burst-rate channel *R* records the per-state five-second peaks. A session in which any channel exceeds its calibrated threshold is declared an attack (Section 4.6), and its rows ranked by $\sigma_{\mathrm{row}}$ attribute the verdict to the most surprising messages (Section 5.7).
**Algorithm 2** Per-session scoring over a single forward pass of the calibrated chain, producing the attack verdict, the per-channel scores, and the ranked per-row attribution**Require:** Session *X* of *M* rows; calibrated chain $\Theta =(S,T,\hat{P},N_{F},S_{\max},S_{\max}^{f})$; channel thresholds 
     $\theta_{T}\left(\cdot \right),\theta_{F},\theta_{R}\left(\cdot \right)$; weights $w_{t},w_{f}$; window size *N*; pooling depth *k*; constraint-field set F
**Ensure:** Verdict y∈{normal,attack}; channel scores (T,F,R); ranked attribution list *A*
  1:**procedure** Score($X,\Theta ,\theta_{T},\theta_{F},\theta_{R},w_{t},w_{f},N,k,F$)  2:      $s_{\mathrm{prev}}\leftarrow \mathrm{undefined}$; F←0  3:      **for** each row r∈X in temporal order, index *m* **do**  4:            s←(r.τ,r.δ,r.μ,r.ν)  5:            $\sigma_{t}\leftarrow$TransitionSurprise$\left(s_{\mathrm{prev}},s,\Theta \right)$                ▹ Equation (3)  6:            $\sigma_{f}^{\max}\leftarrow$FieldRarity $\left(r,s_{\mathrm{prev}},s,\Theta ,F\right)$               ▹ Equations (4)–(7)  7:            $\sigma_{\mathrm{row}}^{\left(m\right)}\leftarrow w_{t}\;\sigma_{t}+w_{f}\;\sigma_{f}^{\max}$                      ▹ Equation (8)  8:            $F\leftarrow \max\left(F,\;\sigma_{f}\;\mathrm{of}\;\mathrm{Equation}\;(6)\;\mathrm{for}\;\mathrm{the}\;\mathrm{ciphering}\text{-}\mathrm{algorithm}\;\mathrm{field}\;\mathrm{of}\;r\right)$  ▹ field channel  9:            update the five-second per-state count for *s*               ▹ rate channel10:           $s_{\mathrm{prev}}\leftarrow s$11:      **end for**12:      partition $\sigma_{\mathrm{row}}^{\left(1..M\right)}$ into ⌈M/N⌉ windows; $\sigma_{\mathrm{win}}\leftarrow$ window means        ▹ Equation (9)13:      T← mean of the *k* largest $\sigma_{\mathrm{win}}$                   ▹ Equation (10)14:      R← per-state peak five-second counts15:      y←attack **if** $T\geq \theta_{T}\left(\mathrm{RAT}\right)$ **or** $F\geq \theta_{F}$ **or** any peak in *R* exceeds $\theta_{R}\left(\mathrm{state}\right)$ **else** normal16:      A← rows of *X* sorted by $\sigma_{\mathrm{row}}^{\left(m\right)}$ in descending order           ▹ attribution17:      **return** (y,(T,F,R),A)18:**end procedure**


## 5. Experiments

### 5.1. Dataset

The evaluation dataset combined normal captures from a commercial cellular network and a controlled testbed with attack captures from two sources. Most attack captures were generated on the authors’ testbed with open5gs and srsRAN, and the remainder were public samples from the FBSDetector dataset of Mubasshir et al. [9]. Both sources were testbed-generated, so no attack capture was taken from a live commercial network. The testbed and commercial captures passed through the MODI on-device preprocessor [26], the FBSDetector pcap samples were dissected separately, and all inputs were aligned to the row schema of Table 2. Captures span LTE and 5G air interfaces and are scored with one verdict per capture session. Nine commercial normal captures were additionally recorded while the device camped on a 3G cell during the commercial collection. Every frame in these captures is a 3G RRC broadcast or paging message that carries no LTE or NR marker, so each row maps to the COMMON network-type value of Section 4.2. These captures contribute benign broadcast-and-paging behavior to the normal calibration set and are scored against the LTE transition threshold of Section 4.6. Acquisition requires root or vendor diagnostic permission on the host device, with the full procedure described in [26].

Each dissected layer-3 message was recorded as a fixed-width row that combines a small set of message-level metadata with selected information elements (IEs) from the NAS and RRC layers of the LTE and 5G control planes. Identifiers follow the 3GPP IE naming used in the relevant specifications (TS 24.301 for NAS-EPS, TS 36.331 for LTE-RRC, TS 38.331 for NR-RRC). Table 2 lists the columns gathered, grouped to match the per-row scoring of Section 4.4, and Table 3 reports the composition of the 192-capture evaluation set, by normal-capture category and by the six FBS attack categories enumerated in Section 2.1.

### 5.2. Evaluation Protocol

Unit of evaluation and partitions.

The unit of classification is the capture session, scored through the 20-row windows of Section 4.5, so no window of a capture can appear on the training side of the partition that evaluates that capture. Three protocols are reported. The deployment configuration calibrates the chain and all thresholds on the full set of 139 normal captures and scores every capture, which matches a device that scores traffic against its own accumulated history of benign signaling. Its normal-side evaluation is in-sample by construction, which is why it is always reported alongside a leakage-free protocol. The leave-one-session-out protocol re-fits the chain and recalibrates every threshold on the remaining 138 normal captures for each held-out normal capture, so no capture influences its own verdict. Attack captures never enter training under any protocol. The cross-validation protocol (CV5) is a stratified grouped five-fold partition at the capture level used for the baseline comparison, in which supervised baselines train on the four training folds with attack labels while the proposed detector uses only the training-fold normals.

Detector configuration.

The proposed detector runs with one frozen configuration in every protocol: smoothing $\alpha =10^{-4}$ and λ=0.1, back-off C=2, window N=20, pooling depth k=3, row weights $w_{t}=0.9$ and $w_{f}=0.1$, and the extreme-value threshold rule of Section 4.6. Nothing is tuned per fold or per protocol. The sensitivity analysis of Section 5.4 varies each of these constants in turn.

Baselines.

Ten lightweight detectors were evaluated under the CV5 protocol. Six supervised classifiers (CatBoost, LightGBM, Random Forest, an MLP with 64 hidden units, a tiny MLP with 16 hidden units, and an N-gram tokenizer feeding XGBoost) trained on the labeled training folds. Four normal-only baselines train on the training-fold normals alone: One-Class SVM and Isolation Forest as point-feature anomaly detectors, DeepLog [34] as an LSTM next-event predictor over the message-token sequence scored by the mean surprise of each observed next token, and LogBERT [35] as a transformer encoder trained with masked log-key prediction and scored by the mean masked-position surprise. The two deep models give the comparison sequence-aware label-free baselines in addition to the point-feature ones. Every baseline scores sessions through the same top-*k* window pooling as the proposed detector and receives the proposed detector’s small-sample fallback threshold rule, the maximum of μ+2σ and the 95th percentile over training-normal session scores. The extreme-value tail estimate of Section 4.6 is the proposed detector’s primary rule, but at this calibration size it rarely accumulates the exceedances needed for a stable fit, so the fallback governs most channels and the baselines are matched on the threshold rule actually in force rather than disadvantaged. All detectors consume the same 17-column dissected records of Table 2.

Metrics.

Each detector emits a binary verdict per session. The harness reports accuracy, precision, recall, true-negative rate, and F1, together with the full confusion-matrix totals (TP, FN, TN, FP). Let TP, FN, TN, FP denote the counts of true positives, false negatives, true negatives, and false positives respectively, where the positive class is attack. The five classification metrics are defined as

(12) $$\mathrm{Accuracy}\;=\;\frac{\mathrm{TP}+\mathrm{TN}}{\mathrm{TP}+\mathrm{TN}+\mathrm{FP}+\mathrm{FN}},$$

(13) $$\mathrm{Precision}\;=\;\frac{\mathrm{TP}}{\mathrm{TP}+\mathrm{FP}},\;\mathrm{Recall}\;=\;\frac{\mathrm{TP}}{\mathrm{TP}+\mathrm{FN}},$$

(14) $$\mathrm{TNR}\;=\;\frac{\mathrm{TN}}{\mathrm{TN}+\mathrm{FP}},\;F_{1}\;=\;\frac{2\cdot \mathrm{Precision}\cdot \mathrm{Recall}}{\mathrm{Precision}+\mathrm{Recall}}.$$

Precision quantifies the share of detector alerts that correspond to genuine attack sessions, which directly reflects analyst-triage cost. Recall quantifies the share of attack sessions the detector flags. The true-negative rate (TNR), also called specificity, is the analogous quantity for normal sessions and is the more informative metric for an on-device monitor that must avoid spurious alerts during long idle periods. F1 is the harmonic mean of precision and recall and serves as the single-number summary used to rank detectors. Uncertainty is quantified with 95% bootstrap confidence intervals (10,000 resamples) on the headline metrics, Wilson 95% intervals on per-category recall, and exact McNemar tests on paired per session CV5 decisions against every baseline. Resource cost is measured with the tiered Docker harness of Section 5.5.

### 5.3. Detection Effectiveness

The evaluation validates the central claim of the title, that the detector identifies false base station attacks. In the deployment configuration the detector reaches an F1 of 96.23% (accuracy 97.92%, precision 96.23%, recall 96.23%, TNR 98.56%, with 51 TP, 2 FN, 137 TN, and 2 FP). Under the leakage-free leave-one-session-out protocol it reaches an F1 of 88.70% with a 95% bootstrap interval of [82.00, 94.31], at recall 96.23% [90.38, 100.00] and TNR 92.09% [87.23, 96.30]. Recall is identical at 96.23% under both protocols because attack captures never enter training, so attack detection does not depend on having seen the scored session. The difference between the two configurations is confined to false positives on the normal side, which Section 6.1 analyzes as a normal-coverage property rather than a detector error. Table 4 aligns the three protocols with the confusion-matrix totals that produce each headline figure.

Table 5 reports the CV5 baseline comparison, in which every capture is tested exactly once by a model that never trained on it. The proposed detector reaches an F1 of 86.21% under this protocol, below its leave-one-session-out figure because each CV5 fold trains on roughly 80% of the normals rather than all but one. The supervised gradient-boosted models lead on F1 (CatBoost 89.32%, XGBoost 88.46%, LightGBM 88.24%), but an exact McNemar test on the paired per session decisions finds none of these leads statistically significant (CatBoost p=0.33, XGBoost p=0.45, LightGBM p=0.45). The proposed detector detects more attacks than any of them, at a recall of 94.34% against 84.91–86.79%. It is also significantly more accurate than the 64-unit MLP (p=0.005), Random Forest (p=0.003), Isolation Forest (p<0.0001), DeepLog (p=0.043), and LogBERT (p=0.001). Among the label-free detectors the chain has the highest F1 by a wide margin. The two deep sequence baselines bracket the failure modes of learned models at this data scale: DeepLog over-generalizes, reaching 100% recall at the cost of 27 false positives, while LogBERT under-trains on the roughly 110 normal sessions available per fold and recovers only 35.85% recall. The supervised models also receive strictly more information than the proposed detector since they train on labeled attacks, so the comparison is conservative toward the chain.

Table 6 is the direct evidence for the FBS-detection claim. It reports the proposed detector’s recall on each of the six FBS attack categories of Section 2.1 at the session level, with Wilson 95% confidence intervals appropriate for the small per-category counts. The attack-side verdicts are identical under the deployment and leave-one-session-out protocols since attacks are always scored by a chain fitted on normals only, so one table serves both. Five of the six categories are detected in full, and the sixth, Security downgrade, reaches 19 of 21. The small categories carry wide intervals, with three categories holding fewer than five captures, so category-level claims rest on the intervals rather than the point estimates.

The two missed attacks are both Security downgrade captures, a ciphered-NAS anomaly variant and a null-encryption variant. Both preserve protocol message order and confine their evidence to field contents, and in the ciphered-NAS case part of that evidence sits inside an encrypted payload the dissector cannot expand. The field channel catches the other 19 downgrade captures, including null-cipher Security Mode Commands, so the residual blind spot is narrow: a downgrade whose distinguishing value is itself rare in the calibration data or hidden from the dissector. These two misses are the deployment and leave-one-session-out result. Under the cross-validation protocol of Table 5, where each fold fits on roughly 80% of the normals, one further Cell and RAN spoofing capture falls below threshold. That single capture is the gap between the 96.23% recall of the deployment and leave-one-session-out protocols and the 94.34% recall on the cross-validation leaderboard. The two leave-one-session-out misses persist across the sensitivity variants of Section 5.4, so they are genuinely hard cases rather than threshold artifacts.

Table 7 and Table 8 decompose the cross-validation verdicts of Table 5 along the dataset composition of Table 3. Table 7 reports the detected count per FBS attack category for every detector. The recall lead of the proposed detector is concentrated in the Security downgrade category, where it detects 19 of 21 captures against 14 to 16 for the supervised models because the dedicated cipher-field channel catches downgrade captures whose message order is clean. DeepLog reaches full recall in every category at the false-positive cost visible in Table 8. Table 8 reports the false-positive count per normal capture category. The false positives of the proposed detector concentrate in the 5G captures. Of its 13 false positives, 9 fall on the 38 5G normals and 4 on the 92 LTE normals, so the smaller 5G population carries most of the false alarms. This matches the normal-coverage analysis of Section 6.1, and broader collection of commercial 5G procedures is the direct path to reducing those false positives. The nine 3G commercial captures draw no false positive from any label-free detector.

### 5.4. Sensitivity Analysis

Table 9 reports a one-at-a-time sensitivity sweep over the constants of the frozen configuration. Each variant is a full leave-one-session-out pass with all other constants held at their reported values, so each row is directly comparable to the reported F1 of 88.70%. The design subsections of Section 4 cite the individual findings where each constant is introduced. Three observations span the table. The largest single effect is the pooling depth, where scoring each session by its single highest window (k=1) costs 5.5 F1 points. Among the threshold-calibration constants the threshold rule matters most, where replacing the extreme-value tail estimate with the plain μ+κσ fallback costs three F1 points, which quantifies the contribution of the tail-based calibration. Recall is 96.23% in 19 of the 24 variants, so detection power is stable and the variation is confined to the false-positive side.

### 5.5. Benchmark Setup

The benchmark runs every detector inside a single pinned Docker image under three device-class CPU budgets enforced through cgroup limits: a constrained tier (1 vCPU, 2 GB), a mid-range tier (2 vCPUs, 4 GB), and a flagship tier (4 vCPUs, 6 GB). Each detector is measured in its own container invocation, so one detector’s allocations cannot inflate another’s measurement. Peak memory is the kernel-reported peak resident set size of the detector process, read through the getrusage interface, and the reported working set is that peak minus a baseline resident set captured after the dataset and all libraries are loaded. Inference cost is the wall-clock time to score one capture session end to end, reported as the median over the 192 sessions, with file parsing warmed out of the timed path. The host is an Intel Core i5-13600K (14 cores, 20 threads) with 64 GB of memory running Ubuntu 24.04 and Docker 29.5, and every library version inside the image is pinned in the benchmark Dockerfile.

### 5.6. Resource Profile

**Figure 6:**
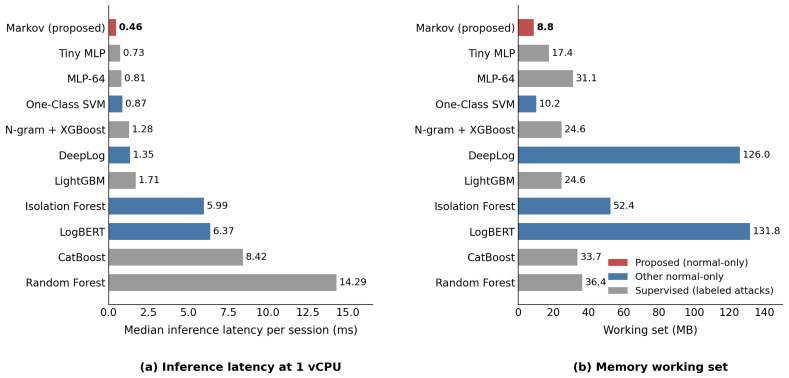
Per-session inference latency at the constrained single-CPU tier (**a**) and detector-attributable working set (**b**) for the proposed detector and ten baselines, sorted by latency and colored by training regime.

Figure 6 compares the detectors on the two axes a hardware-constrained UE-side monitor must respect, the per session inference latency at the constrained single-CPU tier and the detector-attributable working set. The proposed detector has the shortest bar in both panels: it scores a session in 0.46 ms at the median, the fastest of the eleven detectors, within an 8.8 MB working set, the smallest of any detector, and at a serialized model size of 119 KB, second only to the 84 KB tiny MLP. Per-session scoring is bound by single-thread performance, so latency is flat across the 1, 2, and 4 vCPU budgets for every detector except DeepLog and LogBERT, whose medians vary across tiers without improving. The constrained single-CPU budget therefore costs the chain nothing, and for all but those two baselines the constrained-tier latency in the figure is representative of all three tiers. The two deep unsupervised baselines sit in the upper region: DeepLog scores a session in about 1.3 ms within a 126 MB working set and LogBERT in about 6.4 ms within a 132 MB working set, an order of magnitude beyond the chain on the memory axis. The largest serialized models are Random Forest at 9.3 MB and the 64-unit MLP at 5.0 MB, against the chain’s 119 KB, a model-size comparison distinct from the working-set axis of panel (b). Calibration of the chain, a single counting pass plus threshold computation over the training normals, completes in 0.23 s for the constrained tier, faster than the training time of six of the ten baselines.

### 5.7. Per-Row Attribution

Each flagged session is accompanied by a list of contributing rows ordered by per-row score, with the source state, destination state, conditional transition probability, and per-field conditional probabilities reported for each (Section 4.4). On the representative identity-catching capture of Figure 4, the top contributing rows are the repeated pre-security identity interrogation. The row (NAS + RRC, UL, identity_response, LTE) → (NAS + RRC, DL, identity_request, LTE) is flagged as an unseen transition at the pinned surprise of 0.9, and the surrounding interrogation rows rank directly below it, identifying the attack locus and providing the forensic context that an on-device triage tool needs.

## 6. Discussion

The result is not that classical sequence models inherently outperform deep learning on cellular layer-3 data. On this specific task the threat-relevant information lives in the order of a small alphabet of dissected message types, and a model whose representational capacity matches the task complexity is sufficient. The two deep label-free baselines make the point from both sides: DeepLog over-generalizes at this data scale, and LogBERT under-trains, while the supervised gradient-boosted models need labeled attacks to reach an F1 with no statistically significant difference from the chain’s (Table 5). The compact representation translates into a deployment fit no baseline matches in Figure 6: the chain is the fastest detector at every CPU tier with the smallest working set, and its latency is unchanged under the 1 vCPU constrained budget.

Field evidence is layered deliberately. The per-row weight $w_{f}=0.1$ keeps field rarity contributing to the transition channel, and the dedicated field channel of Section 4.6 monitors the ciphering algorithm with its own threshold, which is what catches null-cipher Security Mode Commands whose surrounding sequence is clean. The sensitivity sweep shows the per-row ratio itself is not load-bearing between 0.9/0.1 and 0.7/0.3, while removing field evidence entirely costs four F1 points, so the design conclusion is that field coverage matters and its exact weighting does not.

Pacing illustrates the same design principle in reverse. The state representation of Section 4.2 already excludes message pacing and delegates it to the burst-rate channel. On the present attack set removing that channel leaves every verdict unchanged (Table 9), so the channel is a guard against flood-style pacing attacks rather than a hidden source of the reported recall, and the discriminative power of the transition channel rests on the protocol-identifying four-tuple alone.

The protocol-bounded state also bounds how the model grows, which matters for a long-lived on-device monitor and for extension to future radio generations. The four-tuple vocabularies of three packet types, three directions, 128 message identifiers, and three network types admit 3456 product states, of which only 142 (4.1%) occur in the calibration data. The observed transitions cover 3.1% of the squared state count. The protocol grammar rather than the tuple arithmetic therefore governs the effective state space. The state count saturates quickly, with 107 of the 142 states present after 50 of the 139 calibration captures and only 35 added by the remaining 89. The model stores one entry per observed state and transition, so its memory is O(|S|+|T|) and the serialized size grows linearly, from 13 KB at 10 captures to 116 KB at 139. The 119 KB deployment artifact of Section 5.6 is this serialized chain plus the calibrated channel thresholds. A future radio generation adds one network-type value and one bounded message vocabulary that combine only with each other, so the space grows additively per generation rather than multiplicatively across them, and a message type unseen at calibration maps to the maximum-surprise unknown-state score of Equation (3), which degrades toward conservative flagging rather than silent misses.

### 6.1. Complete Versus Held-Out Normal Calibration

The deployment configuration and the leave-one-session-out protocol bracket the detector’s behavior from two directions, and the gap between them is informative rather than incidental. The attack side is identical for both, so the gap is carried entirely by the normal side: two false positives for the deployment calibration against eleven under leave-one-session-out conditions. Each additional false positive is a normal session whose signaling flows appear in no other normal capture, so when the protocol holds that session out, the chain loses its only training evidence of the behavior and the unseen-transition score rises. The held-out figure therefore measures the completeness of the normal-side coverage of a 139-session calibration set as much as it measures the detector, and the deployment figure shows the same detector once its calibration set contains the behaviors it is asked to judge. The claim this study makes is scoped accordingly: the detector flags signaling that is abnormal relative to its calibrated history, and the false-positive rate on legal-but-rare behavior falls as the calibration history grows. Broader normal collection, particularly of commercial 5G procedures, is the direct path to closing the gap between the two figures.

### 6.2. Limitations

Four bounds qualify the headline numbers. First, every attack capture is testbed-generated rather than captured from a live network (Table 3) because a false base station cannot lawfully be transmitted on one. The set is dominated by categories the testbed reproduces well, and the two missed Security downgrade captures mark the residual blind spot, an adversary who preserves message order and confines its evidence to rare or dissector-hidden fields, which synthetic FBS traces such as the GAN-generated samples of [36] could probe. Second, diversity across operators and Standalone cores is limited. The 101 commercial captures among the 139 normals (Table 3) come predominantly from one operator, so the false-positive behavior on other operators is not yet measured. The 5G Standalone attacks come from one open5gs and srsRAN configuration, so attack-side behavior on alternative cores is untested. Multi-operator normal collection and attacks reproduced on additional open-source stacks are left to the future work of Section 7. Third, the 192-session set yields wide intervals, and at 139 calibration sessions the threshold rule often falls back to its small-sample estimate. Fourth, the capture path requires root and the Qualcomm modem diagnostic interface, which restricts the prototype to rooted Qualcomm-modem devices and excludes stock handsets. This restriction follows from the observation surface rather than the detector, and the prior UE-side monitors PHOENIX [10] and SnoopSnitch [27] share it (Table 1).

## 7. Conclusions

This study presented a lightweight UE-side first-order Markov-chain detector for FBS attacks. The chain and the decision thresholds are calibrated on normal traces alone, with no attack labels at any stage, and the detector fuses information-theoretic transition surprise, conditional field-value rarity, and burst-rate evidence into a per session verdict with per-message attribution. On the 192-capture LTE and 5G evaluation set of Section 5 the detector reaches an F1 of 88.70% under the leakage-free leave-one-session-out protocol and 96.23% in the in-sample deployment calibration, detecting 51 of the 53 attack captures for both. In cross-validation it trails the strongest supervised baselines, which train on labeled attacks, by margins that are not statistically significant. It pairs that accuracy with a 119 KB model, the smallest working set (8.8 MB), and the fastest per session inference (0.46 ms) in the comparison.

Three directions extend the work. (1) Broader normal coverage and cross-operator collection. The gap between the deployment and held-out calibrations is a normal-coverage gap (Section 6.1), and the commercial traffic is drawn predominantly from one operator, so multi-operator field collection directly tightens both the false-positive figure and the generalization claim. (2) Cross-vendor 5G Standalone generalization. The 5G Standalone attack subset rests on a single open5gs and srsRAN testbed (Section 6.2). Replicating the result on alternative open-source Standalone stacks would extend the 5G claim, and running the attacks over the air on licensed private 5G spectrum, such as Korea’s e-Um 5G allocation, or under an experimental-station permit would add radio-channel realism beyond a wired testbed, since transmitting against a commercial network remains unlawful. (3) Deployment integration. Packaging the detector behind the on-device MODI preprocessor [26] and measuring sustained CPU and memory cost under continuous live capture conditions on resource-constrained edge devices is the path to operational evaluation.

## Figures and Tables

**Table 2:** Columns recorded per dissected layer-3 message (5 metadata + 9 scored + 3 unscored fields = 17 columns).

Group	Columns
Metadata (5)	timestamp, packet type, direction, message info, network type
Scored constraint fields (9)	LTE-RRC: cipheringAlgorithm, integrityProtAlgorithm, releaseCause, cellBarred, MCC, MNC, q-RxLevMin. NAS-EPS: EMM cause, identity type
Recorded, unscored fields (3)	NAS-EPS: detach type, ESM cause. GSM-A: AUTN AMF

**Table 3:** Composition of the 192-capture evaluation set by normal-capture category and by attack category.

Normal Capture	Traces	Attack Category	Traces	Source
3G commercial	9	Identity exposure	4	Author, FBSDetector
5G NSA commercial	31	Rejection-based DoS	7	Author, FBSDetector
5G SA testbed	7	Security downgrade and manipulation	21	Author, FBSDetector
LTE commercial	61	Authentication attacks	3	Author, FBSDetector
LTE testbed	31	Cell and RAN spoofing and control-plane hijacking	17	Author, FBSDetector
Total normal	139	Resource exhaustion	1	Author
		Total attack	53	Combined

**Table 4:** Per-session detection metrics of the proposed detector under the deployment, leave-one-session-out, and cross-validation protocols.

Protocol	Training Normals	Acc	Prec	Recall	TNR	F1	TP	FN	TN	FP
Deployment (in-sample on normals)	139 (all)	97.92	96.23	96.23	98.56	96.23	51	2	137	2
Leave-one-session-out	138–139 per fold	93.23	82.26	96.23	92.09	88.70	51	2	128	11
Cross-validation (CV5)	110–112 per fold	91.67	79.37	94.34	90.65	86.21	50	3	126	13

**Table 5:** Per-session detection metrics under the stratified grouped five-fold cross-validation protocol on the 192-capture evaluation set.

Detector	Training	Acc	Prec	Recall	TNR	F1
CatBoost	Normal + attack	94.27	92.00	86.79	97.12	89.32
N-gram + XGBoost	Normal + attack	93.75	90.20	86.79	96.40	88.46
LightGBM	Normal + attack	93.75	91.84	84.91	97.12	88.24
Markov chain (proposed)	Normal only	91.67	79.37	94.34	90.65	86.21
Tiny MLP (16 units)	Normal + attack	88.54	74.60	88.68	88.49	81.03
DeepLog (LSTM)	Normal only	85.94	66.25	100.00	80.58	79.70
Random Forest	Normal + attack	82.81	63.51	88.68	80.58	74.02
MLP (64 units)	Normal + attack	82.81	63.51	88.68	80.58	74.02
One-Class SVM	Normal only	85.94	86.11	58.49	96.40	69.66
LogBERT	Normal only	79.69	79.17	35.85	96.40	49.35
Isolation Forest	Normal only	71.88	0.00	0.00	99.28	0.00

**Table 6:** Per-FBS-attack-category recall of the proposed detector at the session level, with Wilson 95% confidence intervals.

Attack Category	Sessions	TP	FN	Recall (%)	95% CI (%)
Identity exposure	4	4	0	100.00	[51.0, 100.0]
Rejection-based DoS	7	7	0	100.00	[64.6, 100.0]
Security downgrade	21	19	2	90.48	[71.1, 97.3]
Authentication	3	3	0	100.00	[43.8, 100.0]
Cell/RAN spoofing	17	17	0	100.00	[81.6, 100.0]
Resource exhaustion	1	1	0	100.00	[20.7, 100.0]

**Table 7:** Per-FBS-attack-category detected counts for every detector under the five-fold cross-validation protocol.

Detector	Identity	DoS	Downgrade	Auth.	Spoofing	Exhaustion
CatBoost	4/4	7/7	15/21	3/3	16/17	1/1
N-gram + XGBoost	4/4	7/7	14/21	3/3	17/17	1/1
LightGBM	4/4	7/7	14/21	3/3	16/17	1/1
Markov chain (proposed)	4/4	7/7	19/21	3/3	16/17	1/1
Tiny MLP (16 units)	4/4	7/7	16/21	3/3	16/17	1/1
DeepLog (LSTM)	4/4	7/7	21/21	3/3	17/17	1/1
Random Forest	4/4	7/7	15/21	3/3	17/17	1/1
MLP (64 units)	4/4	7/7	16/21	3/3	16/17	1/1
One-Class SVM	0/4	4/7	7/21	2/3	17/17	1/1
LogBERT	0/4	3/7	9/21	2/3	4/17	1/1
Isolation Forest	0/4	0/7	0/21	0/3	0/17	0/1

**Table 8:** False-positive counts per normal capture category for every detector under the five-fold cross-validation protocol.

Detector	3G Commercial	5G NSA Commercial	5G SA Testbed	LTE Commercial	LTE Testbed
CatBoost	0/9	1/31	1/7	1/61	1/31
N-gram + XGBoost	0/9	2/31	1/7	1/61	1/31
LightGBM	0/9	2/31	1/7	0/61	1/31
Markov chain (proposed)	0/9	7/31	2/7	2/61	2/31
Tiny MLP (16 units)	0/9	4/31	3/7	5/61	4/31
DeepLog (LSTM)	0/9	16/31	3/7	7/61	1/31
Random Forest	1/9	5/31	3/7	15/61	3/31
MLP (64 units)	1/9	5/31	2/7	15/61	4/31
One-Class SVM	0/9	3/31	1/7	0/61	1/31
LogBERT	0/9	0/31	5/7	0/61	0/31
Isolation Forest	0/9	0/31	0/7	1/61	0/31

**Table 9:** One-at-a-time sensitivity of the leave-one-session-out F1 to each configuration constant, with the frozen configuration at 88.70%.

Parameter (Frozen Value)	Variants	F1 per Variant (%)
Smoothing α ($10^{-4}$)	$10^{-2},10^{-3},10^{-5},10^{-6}$	88.70 in all four cases
Window *N* (20)	10/30/40/50	85.71/88.70/88.70/88.70
Pooling depth *k* (3)	1/5	83.19/85.47
Row weights $w_{t}/w_{f}$ (0.9/0.1)	0.8/0.2/0.7/0.3/1.0/0.0	88.70/88.70/84.75
Unseen-transition score (0.9)	0.8/1.0	87.72/87.93
Field smoothing λ (0.1)	0.01/1.0	86.21/88.70
Threshold rule (extreme-value tail, u=0.80)	μ+κσ only/u=0.90	85.71/85.71
Transition κ (2.0)	1.5/2.5	87.18/88.70
Rate window (5 s)	removed/2.5 s/10 s	88.70/88.70/87.93

## Data Availability

The dissected capture set used in the experiments is openly available at https://github.com/roastedbeans/modi-dataset (accessed on 17 June 2026). All commercial-network captures were collected on devices and subscriptions operated by the authors. The benchmark harness, including the pinned Dockerfile and the per-detector configurations, is available from the corresponding author upon reasonable request. The MODI preprocessing component is described in [26].
